# The Formation and Function of the VTA Dopamine System

**DOI:** 10.3390/ijms25073875

**Published:** 2024-03-30

**Authors:** Guoqiang Hou, Mei Hao, Jiawen Duan, Ming-Hu Han

**Affiliations:** 1 Faculty of Life and Health Sciences, Shenzhen Institute of Advanced Technology, Chinese Academy of Sciences, Shenzhen 518055, Chinam.hao@siat.ac.cn (M.H.); jiawenduan123@163.com (J.D.); 2 Brain Cognition and Brain Disease Institute, Shenzhen Institute of Advanced Technology, Chinese Academy of Sciences, Shenzhen 518055, China

**Keywords:** dopamine, neurotransmitters, neuropeptides, ion channels, receptors, ventral tegmental area

## Abstract

The midbrain dopamine system is a sophisticated hub that integrates diverse inputs to control multiple physiological functions, including locomotion, motivation, cognition, reward, as well as maternal and reproductive behaviors. Dopamine is a neurotransmitter that binds to G-protein-coupled receptors. Dopamine also works together with other neurotransmitters and various neuropeptides to maintain the balance of synaptic functions. The dysfunction of the dopamine system leads to several conditions, including Parkinson’s disease, Huntington’s disease, major depression, schizophrenia, and drug addiction. The ventral tegmental area (VTA) has been identified as an important relay nucleus that modulates homeostatic plasticity in the midbrain dopamine system. Due to the complexity of synaptic transmissions and input–output connections in the VTA, the structure and function of this crucial brain region are still not fully understood. In this review article, we mainly focus on the cell types, neurotransmitters, neuropeptides, ion channels, receptors, and neural circuits of the VTA dopamine system, with the hope of obtaining new insight into the formation and function of this vital brain region.

## 1. Development of the Dopamine System

Dysfunction of the dopamine system in the mammalian midbrain is implicated in numerous neuropsychiatric and neurological disorders, such as schizophrenia, Parkinson’s disease, and drug addiction. The development and connectivity of dopamine neurons determine their functions in the nervous system. The midbrain dopamine system in the mammalian brain comprises two nuclei, the substantia nigra pars compacta (SNc) and the VTA [1]. Dopamine neurons in the SNc mostly project to the dorsal striatum through the nigrostriatal pathway and regulate voluntary movement [2]. The dysfunction of SNc dopamine neurons induces motor impairment in Parkinson’s disease. Contrarily, VTA dopamine neurons primarily project to the nucleus accumbens (NAc) and prefrontal cortex (PFC) via the mesolimbic and mesocortical pathways, respectively, which play crucial roles in reward and aversion processing, decision-making, and working memory [3,4]. These circuit connections require precise regulation during neurodevelopment.

### 1.1. Ontogeny of the Dopamine System

The midbrain dopamine neurons originate from the ventral midline of the neural tube floor plate [3,5]. Fibroblast growth factor 8 (Fgf8) from the isthmus and sonic hedgehog (Shh) from the notochord induce the production of midbrain dopamine progenitor cells. These progenitors undergo proliferation in the caudal area of the mesencephalon at E8.5–10.5 in the mouse and then migrate to the rostral area at E10.5–12.5. Subsequently, these cells develop axons and dendrites, establishing connections with their targets from E12.5 to birth. This developmental timeline is similar in rat development but occurs two days later than that in mice [6]. Various transcription factors play a guiding role in the early development of dopamine neurons. It is known that the transcription factor Lmx1b maintains *Wnt1* expression [7], which is required for the expression of *Fgf8* and is also essential for the differentiation into specific subtypes of dopamine neurons [8].

Most SNc neurons express *Sox6* (encoding sex-determining region Y (SRY)-related high-mobility-group box 6), and *Sox6*^+^ neurons project to the rostrocaudal region of the dorsal striatum. The SNc to dorsal striatum pathway is important for locomotion [2,9]. Moreover, the substantia nigra pars lateralis (SNpl) to the striatum tail pathway is linked to affective behaviors [10,11]. VTA dopamine neurons mainly project to the NAc and olfactory tubercle in the ventral striatum, the amygdala, and the PFC [12,13], as well as to the lateral septum and entorhinal cortex [11]. Specifically, the VTA to NAc core pathway is involved in acquiring motivational value for a reward, while the VTA to NAc medial shell pathway controls aversion coding [14,15,16].

Human midbrain dopamine neurons are also divided into distinct subtypes according to the expression of different molecular markers. Some of the subtypes bear similarities to those found in the mouse, which includes the expression of *Aldh1a1* (encoding aldehyde dehydrogenase 1 family member A1), *Sox6*, and *Calb1* (encoding calcium-binding protein 1, calbindin 1) in the SNc of the human midbrain [17,18,19]. A human single-cell RNA sequencing (scRNA-seq) study also showed similarities in developmental subsets of midbrain dopamine neurons between human and mouse embryos [20]. However, the overall molecular subdivision of midbrain dopamine neurons in humans, in comparison to rodents, is still not fully understood [4].

### 1.2. The Dopamine System in Invertebrates

Dopamine is an important reward-related neurotransmitter in the brain. However, prior to the year 1957, it was primarily recognized as an intermediary during the norepinephrine (NE) formation [21,22]. With the development of research techniques in biology, it was revealed that dopamine and its receptors constitute a complex system which is capable of controlling multiple behaviors [22]. Importantly, the functions of dopamine heavily depend on the specific type of dopamine receptors present in the target area. Dopamine receptors are one of the family members of G-protein-coupled receptors (GPCRs). In vertebrates, dopamine receptors are divided into two classes: D1 and D2 receptors. D1 receptors activate adenylyl cyclase, leading to an increase in intracellular cyclic adenosine monophosphate (cAMP) levels. On the other hand, D2 receptors oppositely suppress adenylyl cyclase, resulting in a decrease in cellular cAMP accumulation. Additionally, D2 receptors are also involved in modulating the activity of calcium (Ca^2+^) and potassium (K^+^) channels [23]. In invertebrates, dopamine receptors are divided into three groups. The first group (DOP1) resembles the vertebrate D1 receptors, including AmDOP1 from honeybees, CeDOP1 from *C. elegans*, and DmDOP1 from fruit flies. The second group contains AmDOP2 from honeybees and DAMB from fruit flies. Receptors in this group are structurally distinct from the DOP1 but function in the same way as the DOP1, which increases the intracellular cAMP levels when activated by dopamine. The third group is similar to the octopamine receptors in invertebrates, and it is referred to as invertebrate CeDOP2 and CeDOP3 from *C. elegans*, DD2R from fruit flies, and AmDOP3 from honeybees. The activation of these receptors decreases intracellular cAMP levels [24].

Utilizing whole-brain imaging, a recent study on the fruit fly, *Drosophila*, reported that the activity of dopamine neurons in certain brain regions is correlated with locomotion [25]. Another study demonstrated that the dopamine neuron activity in the *Drosophila* brain was correlated with spontaneous fluctuations in walking speed, with some dopamine neurons responding to odors. These dopamine neurons may encode different combinations of locomotion and odor and influence appetitive olfactory behaviors [26]. Furthermore, dopamine signaling plays an important role in regulating sleep and arousal in *Drosophila*. A mutation in the dopamine transporter (DAT) was found to reduce sleep [27], while the activation of dopamine receptors in the dorsal fan-shaped body (dFSB) induces arousal [28]. Moreover, dopamine signaling is also required for learning and memory in *Drosophila* [29]. Fruit flies are capable of learning and remembering aversive and appetitive cues, and dopamine is found to be necessary for acquiring relevant memory [30]. In insects, a brain region known as the mushroom body was shown to regulate sleep/arousal and learning [31,32,33,34]. It still remains unclear whether the same population of dopamine neurons in the mushroom body regulates both sleep and memory. As sleep can promote memory formation [35,36], the fruit fly serves as an excellent animal model for investigating sleep and memory-related mechanisms.

## 2. Features of the Ventral Tegmental Area

In the midbrain, the SNc and the VTA are heterogeneous nuclei in synaptic transmission and connectivity. These two nuclei are widely known for dopamine output and contain dopaminergic, glutamatergic, and GABAergic neurons [37]. The SNc is critical for voluntary movement, whereas the VTA plays complex roles in the stress response, reward- and aversion-related behaviors, and goal-directed behaviors [15]. In the section below, we delved into the structure and function of the VTA in rodents.

### 2.1. Cell Types

Based on previous studies using immunohistochemistry and in situ hybridization, the VTA contains approximately 55–65% of neurons expressing the enzyme tyrosine hydroxylase (TH, for dopamine synthesis); roughly 30% are positive for glutamic acid decarboxylase mRNA (Gad, for GABA synthesis), and 2–3% of cells express vesicular glutamate transporter 2 (Vglut2, for reuptake and packaging of glutamate). The TH^+^ neurons are evenly spread in the VTA, while the GABA neurons diffuse among the dopamine neurons. Vglut2^+^ neurons are densely located in the dorsomedial region of the VTA [38,39,40].

In vitro electrophysiology studies revealed that VTA dopamine neurons exhibit regular pacemaker firings without spontaneous burst activity [41] or GABA_B_ receptor-mediated hyperpolarization [42]. Dopamine neurons are typically identified by hyperpolarization-induced sodium and potassium currents (*I*_h_), which are mediated by hyperpolarization-activated cyclic nucleotide-gated (HCN) non-selective cation channels [43,44]. The *I*_h_ current is a characteristic feature of the dopamine neurons in the lateral VTA and SN but not in the medial VTA [45]. Some GABAergic neurons in the VTA exhibit an *I*_h_ current induced by hyperpolarizing voltage step [42]. Under in vivo conditions, dopamine neurons exhibit both tonic firing and burst firing (also called phasic firing), with an overall firing rate below 10 Hz [44]. However, VTA GABAergic neurons generally exhibit a higher firing rate than dopamine neurons [46,47]. The membrane potential of VTA GABAergic neurons is proven to be insensitive to the GABA_B_ agonist baclofen In vitro [42]. Interestingly, VTA glutamatergic neurons are not easy to identify as their electrophysiological properties are diverse in the distinct subregions of the VTA [38,39,48,49].

In the VTA, dopaminergic, glutamatergic, and GABAergic neurons qualitatively receive similar but quantitatively different patterns of inputs from diverse brain regions [50]. Dopaminergic neurons receive more projections from striatal regions and the globus pallidus, while glutamatergic neurons preferentially receive inputs from the cortical area. In addition, inputs to the VTA GABAergic neurons are primarily from the lateral habenula and laterodorsal tegmental nucleus. Some VTA neurons can co-release other neurotransmitters; a fraction of Vglut2^+^ (glutamatergic) neurons can co-release dopamine or GABA, and a part of TH^+^ (dopaminergic) neurons can also co-release glutamate or GABA [50]. Besides the neurotransmitters, the activities of VTA neurons are also modulated by multiple neuropeptides, including neurotensin, orexin, oxytocin, cholecystokinin (CCK), and corticotropin-releasing factor (CRF). These neurotransmitters, neuropeptides, and their corresponding receptors intricately coordinate and modulate the functions of the VTA.

### 2.2. Ion Channels and Receptors

The ion channels and receptors of VTA dopamine neurons play important roles in processing different information from excitatory and inhibitory inputs. In this section, we discuss the characteristics of ion channels and receptors in the VTA and explain how these channels perform their physiological functions.

#### 2.2.1. Hyperpolarization-Activated Cyclic Nucleotide-Gated (HCN) Channels

There are four HCN subtypes (HCN1-4), all of which are expressed within the VTA of rats [51,52]. Among them, HCN 2-4 channels are highly expressed in midbrain dopamine neurons in mice [53]. The HCN subtypes contain various combinations in different VTA dopamine projection neurons. Studies indicate that the HCN current (usually called *I*_h_) of VTA NAc-projecting dopamine neurons is large and pronounced, whereas the *I*_h_ of VTA PFC-projecting dopamine neurons is relatively small in mice [54,55,56,57].

In a chronic social defeat stress (CSDS) model of depression, a paradigm commonly employed to investigate susceptible and resilient behavioral phenotypes, both the firing rate and *I*_h_ in VTA dopamine neurons were increased in susceptible mice [54]. The local infusion of the HCN channel inhibitor, ZD7288 or DK-AH 269 (also called cilobradine), into the VTA can normalize social avoidance in susceptible mice. These results suggest that the inhibition of *I*_h_ current in VTA dopamine neurons has antidepressant-like behavioral effects in the CSDS model [58]. Recent research demonstrated that the HCN channel inhibitor DK-AH 269 induced ketamine-like rapid and sustained (lasting for 13 days) antidepressant-like effects in susceptible mice [59]. Interestingly, the *I*_h_ of VTA dopamine neurons in resilient animals was even larger than that observed in susceptible mice. This larger *I*_h_ current triggered a compensational increase in potassium (K^+^) currents in resilient animals, which decreased the excitability of VTA dopamine neurons and established homeostatic plasticity in resilient dopamine cells [60]. Lamotrigine (LTG) is an *I*_h_ potentiator, a drug approved by the US Food and Drug Administration (FDA) for the treatment of convulsions and bipolar disorder. A recent study found that the chronic micro-infusion of LTG into the VTA rescued social avoidance in susceptible mice, which is achieved by further increasing the *I*_h_ current and triggering a compensational increase in the K^+^ current—an active “self-tuning” mechanism that was observed in the resilient mice [54]. These results suggest that HCN channels are involved in the regulation of depression-like behaviors and provide a novel bidirectional strategy for the treatment of depression.

#### 2.2.2. Voltage-Gated Potassium Channel Subfamily Q (KCNQ)

KCNQ (K_V_7) channels belong to the family of voltage-gated K^+^ channels and encompass five subtypes (KCNQ1-5). KCNQ2-5 are highly expressed in the central nervous system (CNS) and mediate M-current in various neurons [61], three of which (KCNQ2-4) exist in VTA dopamine neurons [62,63,64]. Studies showed that 92% of NAc-projecting and 96% of PFC-projecting dopamine neurons in the VTA express KCNQ2. In addition, 82% of the NAc-projecting and 83% of the PFC-projecting dopamine neurons are KCNQ3-positive [64].

KCNQ channels can hyperpolarize neurons, stabilize membrane potential, and decrease the excitability of neurons, which links it to conditions such as depression, epilepsy, anxiety, pain, migraine, and drug addiction [65,66,67]. Following CSDS modeling, the susceptible mice demonstrated increased *I*_h_ and the firing rate of VTA NAc-projecting dopamine neurons, along with a trending change in K^+^ currents. Interestingly, the resilient mice exhibited a larger *I*_h_ with significantly enhanced K^+^ currents, which led to a restoration of the firing rate to the control level [54]. The overexpression of KCNQ3 (Kv7.3) channels or local infusion of KCNQ opener ezogabine (also known as retigabine, selective for KCNQ2/3 subunits) in the VTA inhibited the increased firing rate of VTA dopamine neurons in susceptible mice and rescued depression-like behaviors [68]. Fasudil, a KCNQ4 (Kv7.4) activator, also decreased the firing rate of VTA dopamine neurons and demonstrated antidepressant effects in susceptible mice [63]. Tan et al. explored the potential antidepressant efficacy of ezogabine in patients with major depressive disorder and showed that this drug significantly reduced depressive and anhedonic symptoms within these patients [69]. Thus, these studies support that KCNQ channels are a promising target for the treatment of depression as they mimic the active resilience mechanism—a novel therapeutic strategy that is different from the conventional treatments in which the drugs achieve treatment effects by reversing pathological alterations. Importantly, a valuable target revealed from rodent models has the potential to be extrapolated to humans.

#### 2.2.3. Dopamine Receptor and Transporter

Dopamine is a catecholamine neurotransmitter that exerts its effects upon binding to G-protein-coupled receptors [70]. Mammalian dopamine receptors can be divided into two classes, D1 and D2. The D1-like receptor comprises the D1 and D5 subtypes, and the D2-like receptor comprises the D2, D3, and D4 subtypes. The activation of D1-like or D2-like receptors can induce opposing effects on K^+^ currents. D1-like receptor activation reduces K^+^ currents and increases the excitability of the cells, whereas D2-like receptor activation enhances this current and decreases the excitability of cells [71]. D1-like receptors are predominantly distributed in the striatum, NAc, substantia nigra pars reticulata (SNr), olfactory bulb, amygdala, and frontal cortex. On the other hand, D2-like receptors are mostly expressed in the striatum, the lateral part of the globus pallidus, the NAc core, VTA, the hypothalamus, the amygdala, cortical areas, the hippocampus, and the pituitary [70].

The dopamine transporter (DAT) is a transmembrane sodium/chloride-dependent protein selectively expressed in dopaminergic cells. DAT is responsible for the dopamine reuptake from the synaptic cleft and regulates both the extracellular and intracellular concentrations of dopamine [72]. In patients with Parkinson’s disease (PD), DAT density is gradually decreased in the putamen, caudate, and NAc [73]. However, in schizophrenia patients, the level of DAT in the striatum does not change significantly [74,75] despite schizophrenia being a neuropsychiatric disorder induced by striatal hyperdopaminergic activity.

#### 2.2.4. Serotonin Receptor and Transporter

Serotonin (5-hydroxytryptamine, 5-HT) is a monoamine that contributes to the regulation of appetite, sleep, memory and learning, temperature regulation, mood, cardiovascular function, muscle contraction, and endocrine regulation [76]. As an important endogenous neuromodulator in the CNS [77], 5-HT is primarily released by neurons in the dorsal raphe nucleus (DRN) and extensively projected throughout the brain [78]. The dysfunction of 5-HT in the brain may lead to various disorders such as major depression disorder, Parkinson’s disease, obsessive–compulsive disorder, eating disorders, migraine, irritable bowel syndrome, tinnitus, and bipolar disease, each mediated by different 5-HT receptors [76,77].

There are at least 14 subtypes of 5-HT receptors [79], most of which are G-protein-coupled receptors, with the exception of the 5-HT_3_ receptor, which functions as a ligand-gated cation channel [77]. Dopaminergic neurons receive a prominent innervation from 5-HT released by the DRN neurons [80]. 5-HT can induce diverse effects on the activity of midbrain dopaminergic neurons due to the various receptor subtypes. 5-HT neurons innervate both dopamine and non-dopamine neurons in the VTA [81]. These neurons mostly inhibit VTA dopamine neurons while they can also modulate their activities by modifying GABAergic and glutamatergic neurons [80]. On the other hand, the reuptake of serotonin is mediated by the serotonin transporter (SERT) [82]. The SERT regulates 5-HT homeostasis through the Na^+^/Cl^-^-dependent recycling of serotonin into presynaptic neurons [83]. Studies show that selective SERT inhibitors can be used to treat major depression and anxiety disorders, suggesting that serotonin transporters are important therapeutic drug targets [84].

#### 2.2.5. Adrenergic Receptor

Adrenergic receptors (ARs) are members of the large GPCR superfamily and are categorized into three different subunits, namely, alpha1-adrenergic receptors (α1ARs), alpha2-adrenergic receptors (α2ARs) and beta-adrenergic receptors (βARs) [85,86]. Norepinephrine and adrenaline bind to ARs to respond to physiological function. Noradrenergic signaling adjusts VTA dopaminergic activities and cocaine-seeking behaviors during early cocaine withdrawal through α1ARs and α2ARs but not βARs [87]. Acute social defeat stress increased the release of NE and CRF, acting on α1ARs and CRF receptor 2 (CRF-R2) in VTA dopamine neurons, respectively. These elevated the concentration of intracellular calcium (Ca^2+^) via different pathways, changed the synaptic plasticity of VTA dopamine neurons, enhancing cocaine place conditioning [88]. It is worth noticing that α1ARs also modulate glutamate and the GABA synaptic transmission to VTA dopamine neurons during cocaine sensitization [89].

**Figure 1:**
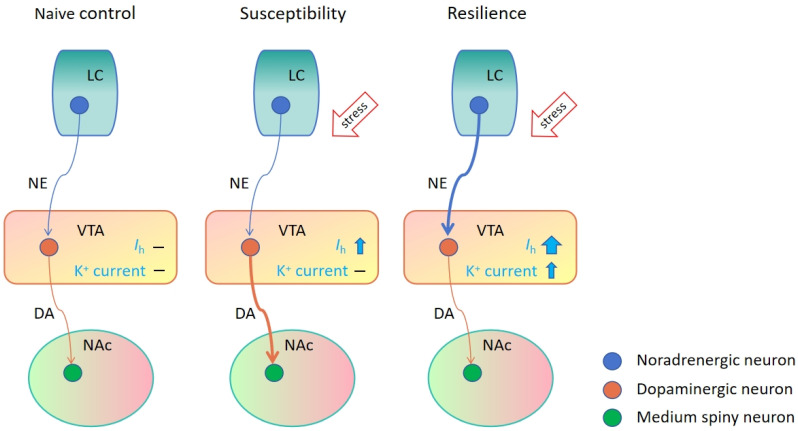
Mechanism of behavioral adaptation induced by chronic social defeat stress. Chronic social defeat stress (CSDS) triggers homeostatic plasticity in the VTA mediated by a balance between excitatory *I*_h_ and inhibitory K^+^ currents. In susceptible mice, CSDS increases the activity of VTA NAc-projecting dopaminergic neurons via the enhancement of *I*_h_, which promotes social avoidance. In resilient mice, CSDS enhances the NE release from the nucleus LC, which leads to an increase in K^+^ currents and greater enhancement of *I*_h_ in the VTA. This adaptive plasticity maintains the control-like activity of the VTA NAc-projecting dopaminergic neurons in resilient mice [54,68,97]. Thus, the LC-VTA-NAc circuit emerges as pivotal for social resilience and anti-depression. NE, norepinephrine; DA, dopamine.

The locus coeruleus (LC) is the main source of NE and contributes to modulating stress and anxiety. The LC projects to VTA dopamine neurons and releases NE, which binds to α- and β-adrenergic receptors in the VTA [90,91,92,93]. The activation of α1ARs in dopaminergic neurons increases *I*_h_ current and decreases small conductance Ca^2+^-activated K^+^ channel (SK) current, which reduces the afterhyperpolarization (AHP) of action potentials and increases the firing rate of VTA dopaminergic neurons [94]. NE neurotransmission in the LC to VTA pathway is required for mediating resilience to social stress [95]. In the CSDS model, VTA-projecting neurons in the LC show enhanced excitatory activity in resilient (but not susceptible) mice. The optogenetic induction of this enhancement reverses depression-like behaviors and the hyperexcitability of VTA NAc-projecting dopaminergic neurons in susceptible mice. A circuit and cell type-specific molecular profiling study illustrated that α1- and β3-ARs were expressed in VTA NAc-projecting dopamine neurons [96,97]. The agonist activation of α1- and β3-adrenergic receptors in the VTA induces effects similar to optical activation, while antagonists blocking these receptors eliminate these effects in previously susceptible mice. The LC-VTA noradrenergic projection and adrenergic receptors are critical for anti-stress (resilience) [97]. Interestingly, following CSDS modeling, the *I*_h_ of VTA dopaminergic neurons is increased in susceptible (depressed) mice, which induced a greater firing rate of VTA dopamine neurons. However, in resilient mice, *I*_h_ and K^+^ currents are also increased, which maintains the firing rate of VTA dopamine neurons at a normal level [54] (Figure 1). The mechanisms by which the LC-VTA NE projection differentially regulates ion channels in susceptible and resilient mice may provide useful information for the development of novel antidepressant treatments.

VTA dopamine neurons have diverse ion channels and receptors for processing and transmitting information to local cells and projection circuits via multiple neurotransmitters and neuropeptides. These channels and receptors are essential for the plasticity of VTA dopamine neurons and eventually promote behavioral adaptation [98,99,100]. It is interesting that the susceptible mice in the CSDS model exhibited increased activity in VTA dopaminergic neurons, manifesting depression-like behaviors. Conversely, in a chronic unpredictable mild stress model, mice showed reduced activity in VTA dopaminergic neurons and depression-like phenotype [101]. Until now, there has been no solid evidence to explain why both increased and decreased activity in these neurons can lead to similar depression-like phenotypes. A plausible explanation is that repeated social defeat stress increased the excitability and baseline firing activity of VTA dopaminergic neurons, which potentially pushes the neuronal activity up toward a saturation point and consequently reduces action potential-induced dopamine release in the target region. A similar scenario is observed in morphine withdrawal conditions, where chronic morphine increased the excitability and the firing rate of the VTA cells but decreased evoked-dopamine release to their target region [102]. Consequently, morphine fails to affect the activity of VTA dopamine neurons during withdrawal (a hyperactive state) [103]. Since dopamine release relies on the inputs from upstream sources, dopaminergic cells with high excitability cannot be regulated normally by inputs, leading to a reduction in dopamine release.

### 2.3. Neuropeptides and Receptors

Neurotransmitters and their receptors in the VTA have diverse functions, and many neuropeptides and their receptors in the VTA are also essential for the modulation of the VTA activities. In this section, we discuss the functions of multiple neuropeptides in the VTA and related pathways.

#### 2.3.1. Corticotropin-Releasing Factor

Corticotropin-releasing factor (CRF) is an important peptide in modulating stress-induced behaviors. Stress induces the release of CRF into the VTA, which subsequently facilitates dopamine release from VTA dopaminergic neurons in specific target regions [104]. CRF is known as a regulator of stress-induced drug-seeking behaviors [105]. CRF-releasing cells are located in the central nucleus of the amygdala (CeA), the bed nucleus of the stria terminalis (BNST), and the paraventricular nucleus of the hypothalamus (PVN) [106]. CRF exerts its influence through its G-protein-coupled receptors, namely CRF receptors 1 and 2 (CRF-R1 and CRF-R2), which activates either the cAMP–protein kinase A (PKA) signaling or the phospholipase C (PLC)–protein kinase C (PKC) pathway [107]. Additionally, CRF-binding protein (CRF-BP) can also bind to CRF and inactivate the needless CRF [108].

CRF in the VTA can affect the activities of dopaminergic neurons. Previous studies found that CRF induces a dose-dependent increase in the firing rate of VTA dopaminergic neurons, which is mediated by CRF-R1 and the PLC-PKC signaling pathway. Interestingly, this effect on the firing increase can be blocked by an *I*_h_ antagonist without changing the voltage dependence of *I*_h_ activation [104]. CRF is released along with glutamatergic projection to the dopaminergic neurons in the VTA [109]. Acute social defeat stress induces an increase in dopamine concentration in the medial PFC (mPFC) and the NAc shell (NAcSh) of rats, and a CRF-R2 antagonist can effectively block the dopamine increase in the NAcSh. After repeated social defeat stress, the CRF-R2 antagonist diminished the dopamine increase in both the mPFC and NAcSh [110]. CRF cooperates with the α1 adrenergic receptor in the VTA, promoting glutamatergic transmission plasticity in the VTA dopaminergic neurons and reinforcing cocaine place conditioning. α1 adrenergic receptors activate the inositol 1,4,5-triphosphate-dependent Ca^2+^ (IP_3_-Ca^2+^) signaling, which can be further enhanced by CRF. Acute social defeat stress induces similar cooperative effects in the VTA [88]. Moreover, the optogenetic activation of the VTA-NAc pathway promotes the release of brain-derived neurotrophic factor (BDNF) in the NAc of socially stressed mice but not in that of stress-naive mice, which is mediated by CRF acting in the NAc [111]. Although investigations on the CRF system received much attention, the precise mechanism underlying CRF modulation in neural circuits is still poorly understood.

#### 2.3.2. Neurotensin

Neurotensin (Nts) is a neuropeptide related to motivated behaviors and regulates the activity of VTA dopaminergic neurons via Nts receptors. One important Nts source in the VTA is the lateral hypothalamus (LH), a brain nucleus that is critical for feeding [112]. It is known that the LH sends feeding-regulating neuropeptides to the VTA. A portion of VTA dopaminergic neurons express neurotensin receptor-1 (Ntsr1). The acute activation of these VTA neurons can decrease food intake, increase locomotor activity, and induce body weight loss in both normal-weight and obese mice. The repeated activation of VTA Ntsr1-expressing neurons induced sustained weight loss over 7 days in obese mice [113]. A recent study revealed that Nts is co-released with other neurotransmitters in the LH-VTA pathway. LH neurons release GABA to GABAergic neurons in the VTA and disinhibit VTA dopaminergic neurons. Moreover, LH neuron terminals can co-release Nts, which act through Ntsr1 expressed in dopaminergic neurons. GABA and Nts coordinate to activate VTA dopaminergic neurons and to promote behavioral reinforcement [114].

#### 2.3.3. Orexin and Dynorphin

LH neurons can co-release orexin (also called hypocretin), and orexin neurons play a role in reward-seeking behaviors through orexin receptor type 1 (oxR1). Orexin neurons can produce orexin A and B, along with the co-release of dynorphin (Dyn) [115]. Dyn is the endogenous ligand of kappa opioid receptors (KORs). Orexin and Dyn have opposing roles in regulating the excitability of VTA dopaminergic neurons and coordinating reward-seeking behavior [116]. Orexin A, when released in the VTA, increases the firing activity in NAc lateral shell- and medial shell-projecting dopaminergic neurons. Conversely, Dyn decreases firing activity in most NAc medial shell- and basolateral amygdala (BLA)-projecting dopaminergic neurons and in a small part of the NAc lateral shell-projecting dopaminergic neurons in the VTA [117]. Furthermore, LH orexin neurons also project to some mPFC-projecting dopaminergic neurons in the VTA [118]. The optogenetic activation of the inputs from the LH orexin and Dyn neurons to the VTA enhances dopamine release in the NAc core, promoting reward-seeking behaviors. This effect can be blocked by oxR1 but not the KOR antagonist, indicating this reward is predominantly mediated by orexin rather than dynorphin [119]. Thus, the outputs of VTA dopaminergic neurons are intricately modulated by the inputs of neurotransmitters and neuropeptides released from upstream nuclei.

#### 2.3.4. Oxytocin

VTA dopaminergic neurons also receive projections from the hypothalamic paraventricular nucleus (PVN). Within this context, PVN neurons release oxytocin (Oxt) in both the VTA and SNc. Oxt enhances the activity of VTA dopaminergic neurons, which regulate social behaviors. However, it is noteworthy that Oxt has a dual effect: it increases the activity of SNc GABAergic neurons, which suppresses the SNc dopaminergic neurons indirectly [120]. Upon activation, Oxt activates G-protein-coupled Oxt receptors (OxtRs) and stimulates the phospholipase C signaling to increase firings [121,122]. In addition, Oxt can also activate vasopressin (Avp) receptors, albeit with lower affinity [123]. Within the VTA, Oxt modulates reward-related behaviors, with the intracranial injection of Oxt resulting in a suppression of sucrose intake [124]. Remarkably, Oxt infusion into the VTA promotes social interactions, whereas the infusion into the SNc inhibits locomotion [125,126]. A recent study examining the effects of social isolation demonstrated that one week of social isolation in adolescent male mice enhances social interaction and induces acute hyperactivity in VTA putative dopaminergic neurons and the sustained expression of GluA2-lacking AMPA receptors. These changes in synaptic plasticity are required for PVN Oxt neurons, suggesting that the PVN-VTA Oxt projection is essential for homeostatic plasticity to modulate social behaviors [127].

#### 2.3.5. Cholecystokinin

Cholecystokinin (CCK) is a gut–brain peptide, and the primary type in the brain is the sulphated carboxy-terminal octapeptide (CCK-8S). Currently, two types of CCK receptors were found, namely, the CCK-A and CCK-B receptors. CCK-A receptors are mainly located in the periphery, while CCK-B receptors are widely distributed in the brain. CCK typically co-exists with dopamine in several mesolimbic dopaminergic neurons. CCK reduces the affinity of dopamine D2 receptors by activating the CCK-B receptors. CCK can also promote the GABA release from the NAc medium spiny neurons (MSNs), possibly resulting from the reduction of D2 receptor activation in the postsynaptic GABAergic MSNs [128]. Recent research revealed that CCK could be released from the somatodendrities of VTA dopaminergic neurons. CCK released in the VTA induces the long-term potentiation (LTP) of GABAergic synapses, which require synaptotagmin-7 and T-type Ca^2+^ channels. Exogenous CCK can also promote LTP, which can be blocked by the CCK2 (CCK-B) receptor antagonist. CCK infusion in the VTA reduces Ca^2+^ signals in dopaminergic neurons and food intake in the overnight-fasted mice. The distance traveled in the open field test was also diminished by the CCK local infusion. Therefore, the peptide CCK can decrease the activities of VTA dopaminergic neurons and influence feeding and locomotion [129].

Together, diverse neuropeptides in the VTA play pivotal roles in multiple behaviors. A summary of the functions of these neuropeptides is shown in Table 1.

## 3. Connectivity of the Ventral Tegmental Area

**Figure 2:**
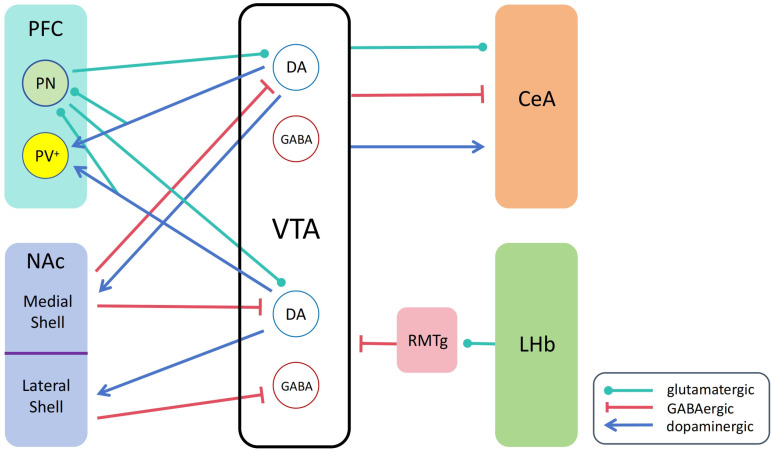
Schematic representation of VTA’s connections with the NAc, PFC, CeA, and LHb. CeA, central nucleus of the amygdala; DA, dopamine; PFC, prefrontal cortex; LHb, lateral habenula; NAc, nucleus accumbens; PN, pyramidal neuron; PV^+^, parvalbumin-positive; RMTg, rostral medial tegmental nucleus; VTA, ventral tegmental area.

The input–output complexity of the VTA neurons determines the diversity of their functions. The VTA receives diverse inputs and innervates multiple nuclei. The targets of the VTA projection are mainly the NAc, PFC, and CeA [130]. In this section, we mainly discuss the reciprocal projection of VTA with the NAc, PFC, and CeA and the inputs from the lateral habenula (LHb) and rostral medial tegmental nucleus (RMTg) (Figure 2). The neurotransmitters and receptors involved in these connections are shown in Table 2.

### 3.1. VTA and NAc

The VTA to the NAc dopaminergic projection constitutes the mesolimbic dopamine system, which is important for motivated behaviors, reinforcement learning, and reward processing. It is known that VTA receives GABAergic projections from the NAc as feedback. In the NAc, the major cell types are GABAergic MSNs, which can be divided into two subtypes according to their expression of D1- or D2-dopamine receptors, and only D1-expressing MSNs project to the VTA [142,143,144,145]. Previous studies showed that neurons in the lateral shell region of the NAc (NAcLat) mainly project to VTA GABA neurons, inducing a disinhibition of dopamine neurons, which projects to the NAcLat. In contrast, the neurons in the medial shell subdivision of the NAc (NAcMed) perform direct inhibitory regulation on two distinct dopamine subpopulations by activating different subtypes of the GABA receptors. NAcMed D1-expressing MSNs inhibit the VTA to NAcMed dopamine neurons via GABA_A_ receptors and inhibit the VTA to NAcLat dopamine neurons via GABA_B_ receptors. This input–output pattern was demonstrated to be critical for regulating motivated behaviors using in vivo optogenetic manipulations [131]. The coordination of opposing effects modulated by distinct NAc subregions underlies the control of complex goal-directed behaviors.

The VTA and NAc are involved in food-induced pleasure, and palatable food preferentially increases dopamine release in the NAc [146,147]. A recent study showed that the activation of VTA dopamine neurons reduces feeding behavior in hungry mice, and refeeding increases the activity of ventral VTA dopamine neurons, especially those of the VTA dopamine neurons that project to the NAc. The food intake reduction induced by the activation of VTA dopaminergic neurons can be diminished by blocking D1 receptors in the NAc or inhibiting the NAc neurons that project to the VTA. These findings suggest that the VTA-NAc circuit is also important for feeding motivation in a hungry state [132]. Besides dopamine and its receptors, neuropeptides also contribute to feeding-related behaviors. Neuropeptides, such as orexin (also called hypocretin) and neurotensin from the hypothalamus project to the VTA neurons, influence the activities of VTA dopaminergic neurons and regulate food intake [146].

### 3.2. VTA and PFC

The projection of VTA dopamine neurons to the PFC is important in drug addiction and mental disorders, including depression. Dopamine release from the VTA may directly induce the activation of PFC parvalbumin-positive (PV^+^) interneurons, and the VTA dopamine neurons can co-release glutamate or GABA in the PFC. A brief activation of the VTA-PFC pathway induces firing activity in the pyramidal neurons and PV^+^ interneurons, which is mediated by glutamate release. Strong stimulation of the VTA gradually induced a prolonged increase in the excitability of PFC PV^+^ interneurons and a short-term enhancement in the excitability of PFC pyramidal neurons. Blocking dopamine receptors or GABA_A_ receptors attenuated these effects, suggesting that dopamine influences the inhibitory transmission in the PFC. The coordination of different neurotransmitter releases could regulate the temporal dynamics of excitatory/inhibitory neurotransmission balance (E/I balance) in the PFC [133].

The PFC can also influence the firing pattern of VTA dopamine neurons. The activation of the PFC increases dopamine levels in the NAc, which can be blocked by glutamate antagonists infused into the VTA (not into the NAc). These observations indicate that PFC neurons can activate dopamine neurons in the VTA. However, glutamatergic neurons in the PFC do not innervate NAc-projecting dopamine neurons directly [134,135]. Most likely, there is another brain nucleus relayed to receive the inputs from the PFC and subsequently release glutamate to the dopamine neurons in the VTA. While most VTA dopamine neurons showed rhythmic bursting induced by stimulation in the PFC, some cells exhibited the opposite pattern. This finding suggests that part of the PFC information is delivered to VTA dopamine neurons via inhibitory relay neurons [148]. These studies underscore the importance of exploring the coordination of the excitatory and inhibitory effects to gain a deeper understanding of the functions of the VTA-PFC circuit.

### 3.3. VTA and CeA

The VTA projects to the CeA directly. The VTA to CeA pathway could be activated during alcohol withdrawal in alcohol-dependent mice and rats. The neurotransmission from the VTA to the CeA is one-third dopaminergic, one-third glutamatergic, and one-third GABAergic [136]. VTA dopaminergic neurons were found to contribute to the reinforcing effects of alcohol in alcohol-preferring rats [137]. Orexin infusion in the VTA of rats enhances saccharin-induced conditioned flavor preference, and the dopamine D1 receptors in the CeA are required for this enhancement [149]. In the VTA of mice, CeA-projecting dopaminergic neurons are involved in pain relief. The decreased expression in the CeA of dopamine D2 receptors is required, and the activation of the D2 receptors in the CeA could reverse this pain relief effect [150]. The VTA-CeA GABAergic neurons were demonstrated to modulate defensive behaviors when faced with a visual threat [138]. Additionally, VTA-CeA glutamatergic neurons can regulate wakefulness and defensive behaviors [139]. Thus, the VTA-CeA pathway is critical for alcohol-seeking, flavor preference, pain relief, wakefulness, and defensive behaviors.

### 3.4. VTA and Lateral Habenula

The lateral habenula (LHb) is a small epithalamic structure that is known for modulating midbrain dopamine neurons [140]. The LHb inhibits VTA dopamine neurons through the glutamatergic activation of GABAergic neurons from the rostromedial tegmental nucleus (RMTg) [141]. The LHb is implicated in motor suppression, cognition, pain, stress, and reward and is important for aversive motivational value and punishment [151]. Chronic stress drives depression via the excitation of the LHb glutamatergic neurons, thereby enhancing the RMTg GABAergic projection to the VTA and leading to the hypoactivity of VTA dopamine neurons [152]. However, part of LHb glutamatergic neurons express aromatic L-amino acid decarboxylase, called D-neurons; acute stress can decrease the excitability of these D-neurons and reduce RMTg GABAergic inputs to the VTA via trace aminergic signaling, leading to the activation of VTA dopamine neurons [153]. The biphasic roles of VTA dopaminergic neurons under stress conditions were found in different stress-induced mouse models. In chronic, unpredictable, mild stress, the VTA dopaminergic neurons exhibit hypoactivity [101], while in CSDS, the VTA dopamine neurons in the susceptible mice show hyperactivity [62]. To date, the mechanism of stress-induced depression still remains controversial.

The LHb neurons dominate VTA dopamine neurons that project to the mPFC and RMTg and modulate aversion in mice [154]. The VTA is known to be important for reward behaviors [46,154]. The LHb and VTA coordinate to regulate aversion and reward behaviors in mice. Most of the LHb neurons are aversion-activated and reward-inhibited neurons, while the majority of VTA neurons are reward-activated and aversion-inhibited neurons [155]. The optogenetic stimulation of the LHb-RMTg-VTA pathway can inhibit VTA dopaminergic neurons and reduce cocaine-seeking behaviors under extinction conditions in rats [156]. Moreover, the LHb can mediate acute nicotine via activating medial VTA while processing chronic nicotine via increasing the activity of lateral VTA [157]. A study in rats using chronic unpredictable mild stress suggests that LHb is a potential therapeutic target for depression treatment [158]. Therefore, the LHb-VTA circuit is essential for the modulation of reward and aversion, drug-seeking, and depression-like behaviors.

## 4. Multiple Neuropsychiatric Disorders

Dysfunction in the dopamine system leads to multiple neuropsychiatric disorders, including Parkinson’s disease (PD), Huntington’s disease (HD), schizophrenia, and major depression. In PD, motor impairment is primarily due to the selective, progressive loss of dopaminergic neurons in the SNc. PD is induced by the degeneration of SNc dopamine neurons but not VTA dopamine neurons, which have a much lower degree of degeneration [159]. Interestingly, these two similar neurons exhibited differential vulnerability in PD. Moreover, HD is also a neurodegenerative disorder characterized by extensive cell death in the basal ganglia, resulting in severe cognitive, motor, and behavioral abnormalities. HD is caused by an expanded polyglutamine-coding (CAG) trinucleotide repeat in the huntingtin (*HTT*) gene, which leads to excitotoxicity, dopaminergic imbalance, mitochondrial dysfunction, metabolic defects, the disruption of proteostasis, transcriptional dysregulation, and neuroinflammation [160]. The dysfunction of dopaminergic transmission is deemed to be responsible for movement-related symptoms, including chorea, rigidity, and akinesia [161].

Schizophrenia is induced by both the degeneration and hyper-regeneration of monoamine axons. The degeneration of monoamine axons produces negative outcomes (social withdrawal and poor rapport) and cognitive symptoms (disorganized thinking and problem-solving). The hyper-regeneration of monoamine axons induces positive symptoms (delusion and hallucination) [162]. In other words, the negative/cognitive symptoms are induced by hypodopaminergic activity, while the positive symptoms arise from hyperdopaminergic activity [163]. In patients with schizophrenia, the dopamine transmission is reduced in the PFC. This induces a cortical hypodopaminergic state, which is associated with impairments in cognitive and executive function. A reduced PFC activity increases dopaminergic projections from the VTA to the striatum through a thalamus–ventral hippocampus (vHipp)-NAc–ventral pallidal (VP) circuit, leading to a hyperdopaminergic state [164]. Major depression is caused by the degeneration of monoamine axons (hypodopaminergic activity) [163]. In depression, the dopaminergic transmission is down-regulated, induced by the hyperactivity of the PFC. The increased excitability of the PFC inhibits the VTA dopaminergic neurons through the enhancement of the BLA-VP pathway and the attenuation of the thalamus–vHipp-NAc-VP pathway [165]. Currently, dopamine D2 receptor antagonists are used to alleviate symptoms in patients with schizophrenia. However, the long-term blockading of D2 receptors will produce D2 super-sensitivity. In the treatment of depression, ketamine can increase AMPA-dependent glutamate transmission and has antidepressant effects with minimal side effects [166,167]. Therefore, more investigations are needed to develop efficient treatments for these neuropsychiatric disorders.

## 5. Conclusions

In this review article, we provide a comprehensive overview of the development of the midbrain dopamine system, with a focus on the construction and connection of the VTA, mostly in rodent models. We also discussed the modulation of VTA activities by multiple neurotransmitters, neuropeptides, and their receptors over the past 5 years. The VTA-NAc circuit has been widely studied, and dopamine release in the NAc is shown to modulate the judgment of importance, reinforcement learning, and processing of aversive stimuli [168,169,170]. The activity of VTA dopamine neurons is heavily regulated by inputs from upstream nuclei. Multiple neurotransmitters, neuropeptides, and their receptors are involved in the modulation of the dopamine system. Analogous to a “manager”, the dopamine system selectively recruits and activates only task-relevant neural pathways as needed. This system guides the animals to perform adaptive behaviors and increases the survival rate [171].

With the development of advanced experimental techniques, we learned more about the midbrain dopamine system. Nevertheless, there are still many questions that persist in this field that need to be answered, for instance, how neurotransmitters and neuropeptides coordinate to perform various functions and how they modulate the activities of ion channels and receptors to maintain the homeostatic plasticity of the dopamine system. Addressing these questions is important for understanding the system in detail and for the potential early diagnosis and treatment of dopamine-related diseases, despite the knowledge obtained from rodent models having various limits in its translation to human beings.

## Figures and Tables

**Table 1:** Functions of neuropeptides in the VTA.

Neuropeptides	Functions	Animals	References
Corticotropin-releasing factor (CRF)	Stress-induced behaviors	Mouse, rat	Tovar-Diaz et al., 2018 [88]; Rodaros et al., 2007 [106]; Walsh et al., 2014 [111];
Neurotensin (Nts)	Feeding inhibition	Mouse	Perez-Bonilla et al., 2021 [113]; Soden et al., 2023 [114];
Orexin	Reward-seeking behaviors	Mouse	Muschamp et al., 2014 [116]; Thomas et al., 2022 [119];
Oxytocin	Social behaviors	Mouse	Musardo et al., 2022 [127];
Cholecystokinin (CCK)	Feeding and locomotion	Mouse	Martinez Damonte et al., 2023 [129];

**Table 2:** Neurotransmitters and receptors in the VTA connections with the PFC, NAc, CeA, and LHb.

Connections	Neurotransmitters and Receptors	References
NAcLat–VTA GABA neurons NAcMed–VTA NAcMed-projecting dopamine neurons NAcMed–VTA NAcLat-projecting dopamine neurons	GABA, GABA receptor GABA, GABA_A_ receptor GABA, GABA_B_ receptor	Yang et al., 2018 [131];
VTA–NAc	Dopamine, dopamine receptor	Cui et al., 2023 [132];
VTA–PFC	Dopamine, dopamine receptor Glutamate, glutamate receptor	Zhong et al., 2020 [133];
PFC–VTA	Glutamate, glutamate receptor	Carr and Sesack, 2000 [134]; Sesack et al., 2003 [135];
VTA–CeA	Dopamine, dopamine receptor GABA, GABA receptor Glutamate, glutamate receptor	Avegno et al., 2021 [136]; Gatto et al., 1994 [137]; Zhou et al., 2019 [138]; Chen et al., 2022 [139];
LHb–RMTg–VTA	Glutamate, glutamate receptor GABA, GABA receptor	Herkenham and Nauta, 1979 [140]; Hong et al., 2011 [141];

NAcLat, NAc lateral shell; NAcMed, NAc medial shell.
